# Clinical outcomes and significance of postoperative ultrasound biomicroscopy in patients with Boston type 1 keratoprosthesis

**DOI:** 10.5935/0004-2749.2023-0160

**Published:** 2024-07-09

**Authors:** Ayse Yildiz Tas, Berk Abay, Orkun Muftuoglu

**Affiliations:** 1 Department of Ophthalmology, Koc Universitesi Hastanesi, Istanbul, Turkey; 2 Department of General Surgery, Barts Health NHS Trust, London, UK

**Keywords:** Boston Keratoprosthesis, Corneal transplantation, Ultrasound biomicroscopy, Anterior segment, Prostheses and implants

## Abstract

**Purpose:**

To determine the clinical outcomes in patients after type 1 Boston keratoprosthesis surgery and the significance of ultrasound biomicroscopy imaging for postoperative follow-up.

**Methods:**

This retrospective analysis included 20 eyes of 19 patients who underwent corneal transplantation with type 1 Boston keratoprosthesis between April 2014 and December 2021. Data on patient demographics, preoperative diagnosis, visual acuity, and postoperative clinical findings were analyzed.

**Results:**

Type 1 Boston keratoprosthesis implantation resulted in intermediate- and long-term positive outcomes. However, blindness and other serious complications such as glaucoma, retroprosthetic membrane formation, endophthalmitis, or retinal detachment also occurred. The use of ultrasound biomicroscopy imaging allowed for better evaluation of the back of the titanium plate, anterior segment structures, and the relationship of the prosthesis with surrounding tissues, which provided valuable postoperative information.

**Conclusion:**

Regular lifetime monitoring and treatment are necessary in patients who undergo Boston type 1 keratoprosthesis implantation for high-risk corneal transplantation. ultrasound biomicroscopy imaging can be a valuable imaging technique for the evaluation of patients with Boston type 1 keratoprosthesis, providing important information on anterior segment anatomy and potential complications. Further studies and consensus on postoperative follow-up protocols are required to optimize the management of patients with Boston type 1 keratoprosthesis.

## INTRODUCTION

According to the World Health Organization, appro­ximately 4.9 million people in the world develop blindness due to corneal pathologies, which accounts for approximately 12% of all blindness cases^(1)^. The most common treatment for corneal opacity is penetrating keratoplasty (corneal transplantation). According to the 2018 report of the Eye Bank Association of America, endothelial failure is the most common indication for keratoplasty in the United States; keratoconus and keratitis are the common causes in other countries^(2,3)^. Recurrent graft failure is observed in conditions such as ocular cicatricial pemphigoid, Stevens-Johnson syndrome, severe chemical burns, and limbal stem cell deficiency. The prognosis of standard corneal transplantations in these cases is poor. The 15-year graft survival rate is reportedly 46% in penetrating keratoplasty and 42% in lamellar keratoplasty^(2)^.

Strampelli et al. first developed the osteo-odonto keratoprosthesis technique in 1963 when searching for a solution for the recurrent corneal rejection of standard transplantations^(4)^. Keratoprosthesis, known as artificial cornea, has been modified over the years (Falcinelli et al., De La Paz et al., Stoiber et al. and Liu et al.), and different methods have been developed^(5,6,7,8)^. The application of type 1 Boston keratoprosthesis (BKPro) with a titanium backplate is currently the most preferred technique. Claes Dohlman developed the BKPro in Massachusetts, which received FDA approval in 1992. According to the January 2019 data, approximately 19,000 BKPros have been used worldwide over the last 20 years (Chodosh J. FDA approval obtained for the Boston Keratoprosthesis type I Lucia design. BOSTON KPro news; July 2019). The BKPro has a collar button design, consisting of a front plate with an optical stem, a corneal allograft button, and a back plate. The front plate is made of medical grade polymethylmethacrylate (PMMA). The radius of curvature of the optical surface, which is 3.5-3.7 mm in central diameter and 5 mm including the front plate, determines the power of the BKPro. The BKPro is available in a single standard pseudophakic power or customized aphakic power for various axial lengths (range: 16-31 mm in increments of 1 mm)^(9)^.

Postoperative follow-up of keratoprosthesis cases is critical. Despite successful surgeries that have been refined recently and the preservation of anatomical integrity, serious complications have been reported^(10,11)^. Glaucoma, retroprosthetic membrane (RPM) formation, endophthalmitis, and retinal detachment are important complications that can be observed in patients with a BKPro. RPM formation is the most common postoperative complication of keratoprosthesis implantation, with an incidence of 25%-65%^(11,12,13)^. The Nd:YAG laser is usually adequate to clear the visually significant membrane. In patients with very thick membranes, surgical excision through the pars plana approach is often required. This membrane is thought to form because of the proliferation of fibrovascular tissue, and the onset is assumed to be multifactorial, involving device-triggered pathologic wound healing as well as host-specific factors^(14)^. Slit lamp biomicroscopic examination is insufficient in these patients. The back of the titanium plate cannot be discerned, and the anterior segment structures cannot be evaluated. To date, there is no standard process other than slit lamp examination for observing the anterior segment and angle anatomy of the eyes implanted with keratoprosthesis. Furthermore, currently, there is no consensus on how to perform postoperative follow-ups in patients undergoing keratoprosthesis.

There are two state-of-the-art diagnostic techniques available for imaging and documenting devices implanted on the cornea. One is the relatively new noncontact method of anterior segment optical coherence tomography (AS-OCT), and the other is the water-immersion technique of ultrasound biomicroscopy (UBM), which uses 35-50 MHz of high-frequency ultrasound waves^(15,16)^. UBM allows the evaluation of the back of the titanium plate, anterior segment structures, and the relationship of the prosthesis with the surrounding tissues. In recent years, UBM has been used in the evaluation of glaucoma, malignant glaucoma, and ciliary body and angle pathologies. In our clinic, some patients were also evaluated using UBM following keratoprosthesis.

The intermediate- and long-term outcomes of BKPro are good. However, the risk of blinding complications after implantation persists, making regular lifetime monitoring and treatment a must. Therefore, we aimed to retrospectively evaluate the postoperative clinical findings in patients from our clinic in whom type 1 BKPro was used for high-risk corneal transplantation.

## METHODS

### Study design

This was a retrospective, consecutive, nonrandomized, interventional case series.

### Patients

Twenty eyes of 19 patients who underwent implantation with a type 1 BKPro between April 2014 and December 2021 were included. All procedures were performed by one corneal surgeon (O.M.). The following patient data were recorded: age, sex, preoperative diagnosis, visual acuity values (Snellen), and slit lamp biomicroscopic examination findings. UBM (Eyecubed Ellex) was performed by the same experienced specialist in 14 of the 20 eyes.

The UBM of all the patients was performed by the same experienced ophthalmologist. The patients were placed in the supine position. Topical anesthesia (0.5% proparacaine HCl) was applied to the eye for imaging, and an eye speculum was inserted. The device’s probe was placed in a transparent sheath filled with 5 ml of 0.9% saline and placed on the corneal surface for imaging. Images were acquired radially and horizontally at the central cornea and subsequently, through 360 degrees perpendicular and horizontal to the limbus. The anterior chamber structures, iridocorneal angle, ciliary processes, presence of RPM, current status of intraocular lens (IOL), and Ahmed glaucoma valve (AGV) tube status were assessed and recorded.

The data are reported as a case series because there is insufficient data to perform statistical analyses.

### Surgical technique

**Figure 1:**
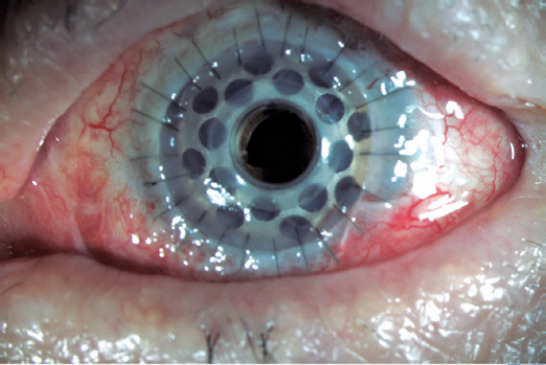
Implanted Boston type 1 keratoprosthesis in a patient with corneal opacity secondary to chemical injury.

The technique for implanting a type I BKPro has been previously described by Dohlman et al.^(17)^. A corneal donor button is prepared (8.5-9.0 mm), and a central 3-mm hole is trephined. For better BKPro centration, the 3-mm central trephination can be performed before the outer diameter punch is used. Thereafter, the donor button is placed over the stem of the front plate, and the back plate is placed on top of the complex. Subsequently, a titanium locking ring is snapped into place. The recipient cornea is prepared as for traditional penetrating keratoplasty, with the host trephine measuring 0.5 mm less in diameter than the donor graft. Finally, the donor button was sutured with multiple interrupted 10-0 nylon stitches (Figure 1).

## RESULTS

Twenty eyes of 16 male (84.21%) and three female (15.78%) patients with a mean age of 49.3 years (range: 32-81) were evaluated. The patients were followed up for 6-52 months (mean: 17.3). The preoperative diagnoses were as follows: 12 eyes of 11 patients had corneal chemical burns, five eyes of five patients had recurrent graft failure, and three eyes of three patients had posttraumatic total vascularized corneas.

Corrected visual acuity on the Snellen chart was 0.3 (3 mps - 0.5). In addition to ocular surface problems, five of the eyes (25%) had eyelid problems such as entro­pion, symblepharon, and lagophthalmos. In the slit lamp examination, AGV was applied to 13 eyes (65%) and not applied to 7 eyes (35%) (Table 1).

**Table 1:** Preoperative diagnoses, UBM observations and postoperative follow-up observations

Preoperative diagnosis	Number of eyes (n=20)	Percentage
Preoperative diagnosis		
**Corneal chemical burn**	12	60
**Recurrent graft failure**	5	25
**Total vascularized cornea following trauma**	3	15
**UBM observations**		
**UBM observation**	**Number of eyes (n=20)**	**Percentage**
**Retroprosthetic membrane**	3	15
**IOL haptics**	3	15
**UBM observations according to AGV tube status**		
**UBM observations**	**Eyes with AGV (n=13)**	**Eyes without AGV (n=7)**
**Significant angle narrowing (%)**	0	4 (57.1)
**Angle narrowing and iridocorneal adhesions (%)**	3 (23)	0
**Tube tip (%)**	4 (30)	0
Postoperative follow-up observations		
**Observation**	**Number of eyes (n=13)**	**Percentage**
**Endophthalmitis**	2	10
**Corneal melting**	6	30
**Cystoid macular edema**	4	10

**Figure 2:**
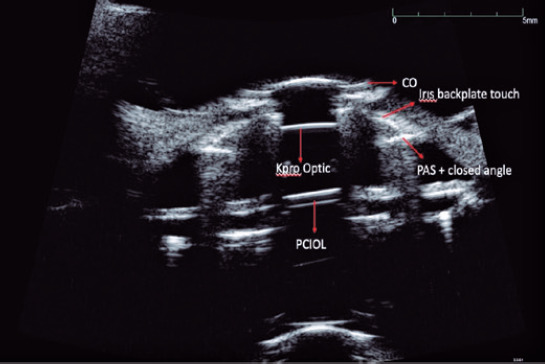
Cross-sectional UBM scan of the eye depicted in Figure 1 showing the assembled BKPro at the apical center of the cornea.

The anterior and posterior plates were viewed using UBM in 14 cases (Figure 2). RPMs were observed in three eyes (15%), and IOL haptics were viewed in three eyes. Significant angle narrowing was observed in four (57.1%) of the seven eyes without an AGV. However, three (23%) of the 13 eyes with an AGV demonstrated angle narrowing and iridocorneal adhesions. The tube tip was visualized in four patients (30.7%) who underwent AGV implantation with UBM (Table 1).

Endophthalmitis was observed in two patients (10%) during the postoperative follow-up. These patients underwent 25 G pars plana vitrectomy and were administered intravitreal antibiotic therapy. Corneal melting was detected in six of the 20 evaluated eyes (30%); scleral patch grafts were placed in two of these eyes (33.3%). Cystoid macular edema was observed in four eyes (20%), and two patients (10%) received intravitreal anti-VEGF injections. None of the patients required prosthesis removal during follow-up.

## DISCUSSION

Type 1 BKPro with a titanium backplate has become a successful treatment for recurrent corneal graft failures in recent years. Over the last decade, the frequency of keratoprosthesis implantation has increased gradually. Despite successful surgeries and preservation of anatomical integrity over time, serious complications are observed. The postoperative follow-up of keratoprosthesis cases is critical.Slit lamp biomicroscopic examinations is not sufficient for evaluating anterior segment structures following keratoprosthesis because the back of the titanium plate cannot be discerned. Occasionally, additional surgery may be required after keratoprosthesis because of the development of glaucoma or lens complications. In cases where multiple surgeries are required, imaging methods can help plan the surgical strategy and predict intraoperative difficulties.

AS-OCT can help evaluate the anterior segment anatomy after KPro implantation and is an important imaging modality ^(18,19)^. AS-OCT is used to visualize the donor-recipient corneal interface, corneal graft, and angle status, and it enables early detection of known complications of KPro implantation^(20)^.

UBM has been an important tool in the diagnosis, evaluation, and follow-up of patients with glaucoma since it was first described as an imaging method with clinical importance in 1992 by Pavlin and Foster^(21)^. Our study shows that UBM can also be an important tool for follow-up evaluation after keratoprosthesis as it can detect angle narrowing, RPMs, and IOL haptics. To the best of our knowledge, there is no study that reports the results of UBM for the follow-up of patients after keratoprosthesis.

In our study, 30% of the patients developed corneal melting postoperatively. Aravena et al. reported a persistent corneal epithelial defect of 43% and a sterile keratolysis rate of 26%^(22)^. In the study by Lee et al., the incidence of corneal melting was 2.4%-30.4%^(23,24)^.

RPM formation is a complication that can develop within a few months, usually during the postoperative period^(25)^. Silva et al. detected RPM formation in 63% of the eyes in their study of 11 eyes using AS-OCT^(18)^. Another significant finding of this study was that all patients with sterile corneal necrosis (melting) had an RPM. Sivaraman et al.^(26)^ also found similar outcomes; AS-OCT revealed backplate RPM formation in all eyes with periprosthesis melting and in 34.1% of eyes without it. In our study, RPMs were observed on UBM in three eyes (3/20 15%). Arevena et al. reported that the incidence of RPMs was 52% in the 5-year follow-up^(22)^. Shapiro et al. reported that the incidence of RPMs on AS-OCT was 77%^(20)^.

Glaucoma is the leading cause of permanent visual loss following BKPro implantation^(27)^. The prevalence of preexisting glaucoma varies from 33.3% to 89.3%^(28)^. Lekhanont et al. reported 90% improvement in vision after keratoprosthesis in their patients, which decreased to 55% within 6 years. The most important reason for this decrease is the lack of any intervention for glaucoma^(29)^. In the study by Gu et al., at 18 months, the IOP in the patients who underwent AGV implantation and the controls was 17.3 ± 5.6 mmHg and 24.6 ± 1.7 mmHg, respectively.^(30)^. In our study, AGV was implanted in 13 patients.

Endophthalmitis is a serious complication that can occur after BKPro transplantation. Chhablani et al. reported that the incidence of endophthalmitis was 3.67% (5/136 eyes), which developed over a mean of 5.62 months after BKPro surgery^(31)^. Endophthalmitis refers to inflammation and infection of the inner layers of the eye, including the vitreous humor and the retina, and it can cause severe visual loss or even blindness if not promptly and appropriately managed. Several risk factors have been identified for the development of endophthalmitis after keratoprosthesis. These include a previous ocular burn, infectious keratitis, corneal melting, and postoperative contact lens wear, which may increase the risk of bacterial colonization and subsequent infection^(32)^.

In conclusion, BKPro is the most commonly used artificial cornea. The evolution of its design over the past two decades not only improved outcomes but also expanded its indications. Although the early and mid­term results are good, there may be serious long-term complications. Thus, follow-ups are critical. In these patients, the management of complications is of great importance. Current efforts are focused on increasing the accessibility of the device. Further studies directed toward improving biointegration and IOP monitoring, among other areas, following keratoprosthesis will hopefully result in better long-term outcomes and a greater therapeutic potential in corneal blindness.
